# Prophylactic Aminophylline for Prevention of Apnea at Higher-Risk Preterm Neonates

**DOI:** 10.5812/ircmj.12559

**Published:** 2014-08-05

**Authors:** Amir Mohammad Armanian, Zohreh Badiee, Raha Afghari, Nima Salehimehr, Akbar Hassanzade, Soghra Sheikhzadeh, Maryam Sharif Tehrani, Gohar Rezvan

**Affiliations:** 1 Department of Pediatrics, Child Growth and Development Research Center, Isfahan University of Medical Sciences, Isfahan, IR Iran; 2 Isfahan University of Medical Sciences, Isfahan, IR Iran; 3 Al-Mahdi University, Isfahan, IR Iran; 4 Department of Epidemiology and Biostatistics, School of Health, Isfahan University of Medical Sciences, Isfahan, IR Iran; 5 NICU Ward, Al-Zahra Hospital, Isfahan University of Medical Sciences, Isfahan, IR Iran

**Keywords:** Apnea of Prematurity, Premature Infant, Methyl xanthine Therapy, Aminophylline

## Abstract

**Background:**

A few studies have been carried on preventive drugs for apnea of preterm neonates.

**Objectives:**

This study aimed to assess the safety and prophylactic effects of aminophylline on the incidence of apnea in premature neonates.

**Patients and Methods:**

This study was a randomized clinical trial (RCT) research. The prophylactic effect of aminophylline on apnea was investigated in premature babies in our NICU (IRAN-Isfahan). In the study group (A), 5 mg/kg aminophylline was initially administered as a loading dose. Then, every 8 hours, 1.5 mg/kg was given as maintenance dose for the next 10 days. In the control group (C), no aminophylline was used during the first ten days of life.

**Results:**

Fifty-two neonates were randomized for the study and all of them completed it. Primary outcomes were clearly different between the two groups. Only 2 infants (7.7%) who had been placed in aminophylline group developed apnea, as compared to 16 infants (61.5%) in the control group (P < 0.001). Three and four neonates (11.5%, 15.4%) in the aminophylline group developed bradycardia and cyanosis respectively, as compared to 16 infants (61.5%) who did not receive aminophylline (P < 0.001). Median time of need to NCPAP (Nasal Continuous Positive Airway Pressure) was 1 (0 - 4) days and 2.5 (0.5 - 6.5) days in group A and C, respectively (P = 0.03). No side effects were reported in neonates (P > 0.999). Median time of hospitalization was shorter in aminophylline group (P = 0.04).

**Conclusions:**

This study supports the preventative effects of aminophylline on apnea in extreme premature infants. In other words, the more premature an infant, the greater the preventative effect of aminophylline on the incidence of apnea and bradycardia.

## 1. Background

Recurrent periods of apnea are common in preterm neonates (1, 2). According to definition of American Academy of Pediatrics (AAP), apnea is a pause in breathing for more than 20 s or less than 20 s accompanied with bradycardia or/and cyanosis (2-4). Prevalence of apnea increases with decrease in gestational age (5, 6). Although apnea can occur spontaneously and only be attributable to the prematurity, but it can also be stimulated or intensified with deprivation of oxygen, metabolic disorder, intracranial pathology, and infection (1).

If apnea gets prolonged, it can lead to hypoxemia or bradycardia in such a way that active resuscitation of the neonate will be needed (1). Clinical concerns exist about harmful effects of apnea on the development of brain or dysfunction of intestine and other organs (5).

Recurrent periods of apnea can be so severe that lead to respiratory failure, which needs intubation and use of mechanical ventilation (MV) (1, 6). Furthermore, it has been shown that apnea and hypoxemia is associated with EEG abnormalities and maybe the cause of leukomalacia, which can lead to subsequent mental and neurological problems (7). Preterm neonates are routinely monitored with pulse oximeter and heart monitoring since 1980 (7). Sudden decrease in O2 saturation (SO2) related or not related to apnea is common in preterm neonates, which needs immediate intervention (2, 8).

One of the old effective therapies for apnea of prematurity (AOP) is using methylxanthines (9). It is believed that methylxanthines can stimulate breathing. Thus they were used for the treatment of apnea since 1970’s (1). Two effective forms of methylxanthines that have been used for recurrent periods of neonatal apnea are theophylline and caffeine (5). Exact mechanism of their action is unknown, but they may trigger chemoreceptors respond to CO2 accumulation; improve the performance of respiratory muscles or stimulate the CNS (1). Caffeine has a similar effect as theophylline (9) but additional therapeutic advantages exist such as large difference between therapeutic and toxic blood level, better intestinal absorption, and longer half-life ( can be used once a day) (9, 10). Methylxanthines can decrease use of mechanical ventilation (MV) (11) and can also be useful before extubation (12).

Avoidance of repeated apnea and hypoxic episodes decreases the O2 administration risk, intubation and other harmful complications (3-5, 7, 9-14). Hence, various studies and measures for the treatment of neonatal apnea have been tried (1, 10). However, a few studies have been done about preventive drugs for apnea in preterm infants (1).

In studies of Bucher et al. and Levitt et al., short-term effects of prophylactic methylxanthines and related signs were investigated (7, 14). They found that there was no obvious difference between the methylxanthines given group and the placebo group regarding the incidence of apnea and short-term outcomes (2, 7, 13). Henderson-Smart DJ et al. in a Cochrane review article reviewed studies on the preventive effects of methylxanthines. They concluded that future studies are needed to examine the effects of prophylactic methylxanthines in preterm infants at higher-risk of apnea (1). 

## 2. Objectives

Given the importance of the prevention of apnea in preterm neonates and based on the conclusions and recommendations of Cochrane review article, we decided to study the prophylactic effects of aminophylline on the incidence of apnea at higher-risk neonates (preterm neonate with a weight below 1200 g).

## 3. Patients and Methods

### 3.1. Study Design and Participants 

The study was a randomized clinical trial (RCT) one. Infants born between March 2012 and April 2013 with a gestational age (GA) < 32 wk, and/or birth weight (BW) < 1200 g, who were admitted to the level 3 neonatal intensive care units (NICU) of the Isfahan University of Medical Sciences at Alzahra and Shahid Beheshti hospitals, were eligible for the participation in the study. Exclusion criteria were major congenital anomalies, infants with a GA > 34 weeks (even with BW < 1200 g), asphyxia, occurrence of apnea and need to mechanical ventilation in the first 24 hours of birth, congenital cyanotic heart disease and sepsis in the first seven days of birth.

The prophylactic effect of aminophylline on the incidence of chronic lung disease (CLD) was investigated in two groups of aminophylline (group A) and control (group C). We used unequal randomization for this trial. The infants in the study were randomly assigned to aminophylline (group A) and no aminophylline (group C) groups by an impartial employee, as described below. In order to select randomly the neonates, those with an even digit at the end of their file numbers were placed in group A and neonates with their file numbers ending in an odd digit were assigned to group C. Care providers were not blinded to an infant’s protocol.

### 3.2. Intervention

In group (A), 5 mg/kg of aminophylline, as loading dose, was begun (parenteral) then every 8 hours, 1.5 mg/kg, as the maintenance dose, was administered for the first 10 days of life.

In group (C), no aminophylline was given in the first 10 days of life. The primary outcome of the study in both groups was the preventative effect of aminophylline on the incidence of apnea, bradycardia and cyanosis during admission in the NICU. Apnea was considered a pause in breathing for more than 20 s or less than 20 s when accompanied with bradycardia (HR < 90) or/and cyanosis (oxygen saturation < 85%) (2-4). These events were detected by team physicians, evaluation of nursing, documentation, and downloading data of calibrated pulse oximetry monitors. Secondary outcomes included continuous positive airway pressure (CPAP) or mechanical ventilation need, death, and also other drug side effects (tachycardia, hypertension) for each neonate, which were recorded daily.

The criteria for intubation and mechanical ventilation composed of frequent apnea (> 3 episodes per hour) associated with bradycardia or a single episode of apnea that required bag and mask ventilation.

### 3.3. Ethical Consideration

This paper is derived from a residency thesis, No.391323 in Isfahan University of Medical Sciences. The study was approved by the regional Ethics Review Board. Written informed consent was obtained from parents. This trial was registered at www.irct.ir with number ID: IRCT2013052610026N1. All authors had no conflicts of interest to disclose.

### 3.4. Data Analyses

The sample size of 52 infants was based on the sample size design for the primary outcome of a previous study (the effect of prophylactic methylxanthines on prevention of apnea in preterm infants) (13). The sample size was adjusted according to the opinion of a statistical consultant. Normally distributed and nonparametric data were presented by means (± SD) and median (IQR). The results were compared with the chi-square, independent t test, Mann-Whitney, and Fisher exact test. The data was analyzed by using the SPSS statistical software version 20.

## 4. Results

**Figure 1.**
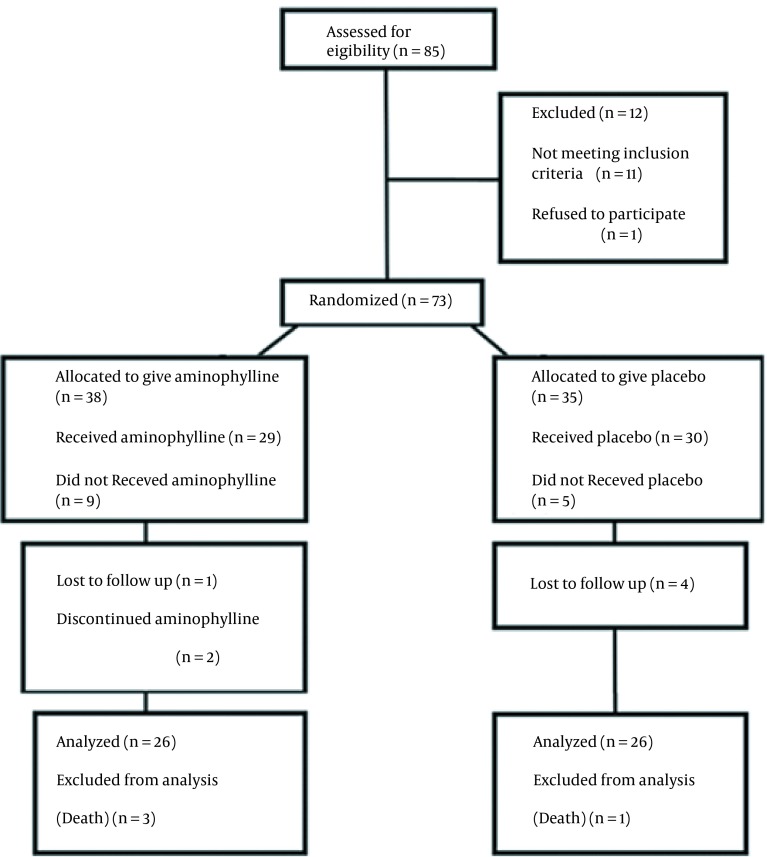
Consort Diagram Showing the Flow of Samples Through Each Stage of Study.

In our study, 52 neonates were randomized and completed the study (Figure 1), and the results were analyzed with the intention of health promotion and treatment. Demographic characteristics were similar between the two groups (Table 1). Primary outcomes were clearly different between two groups. Two infants (7.7%) in the aminophylline group developed apnea, as compared to 16 infants (61.5%) in the control group (P < 0.001; Table 2). Three and four neonates (11.5%, 15.4%) in the aminophylline group developed bradycardia and cyanosis respectively, as compared to 16 infants (61.5%) in the control group (P < 0.001; Table 2).

Twenty-one neonates in group A and the same number in group C were affected by mild to moderate Respiratory Distress Syndrome (RDS). Average gestational age in group A was 29.89 ± 1.93 wk, and in group C was 28.59 ± 2.06 wk. Average birth weights in group A were 1071.54 ± 117.56 g and in group C were 1007.69 ± 134.02 g, which means the average gestational age and birth weight were similar in two groups (P = 0.10 , P = 0.07, respectively; Table 1). Secondary outcomes such as need to NCPAP (Nasal Continuous Positive Airway Pressure) and mechanical ventilation were investigated. Median time of need to NCPAP in group A was 1 (0 - 4) days, and in group C was 2.5 (0.5 - 6.5) days (P = 0.03; Table 2) that seems to be a bit shorter in group A.

It was only one baby who developed severe apnea and needed mechanical ventilation on the third day of birth (in group A) (P = 0.50). No side effects were reported in neonates (P > 0.999). Median time of hospitalization was shorter in the aminophylline group (P = 0.04, Table 2); median time of hospital stay was 32.5 d (23 - 53) and 41 d (27.5 - 50.5) in groups A and C respectively.

Three neonates who died in group A had BW of 830, 1170, 1100 g and GA of 30, 32, 29 wk, respectively. These babies died at 14, 12, 10 days of their birth, respectively because of late onset sepsis. Only one baby died in group C (BW: 970 g and GA: 29 wk). He died on 18^th^ day of birth (P = 0.31).

The incidence of PDA (Patent Ductus Arteriosus), IVH (Intraventricular Hemorrhage) and NEC (Necrotizing Enterocolitis) were similar between the two groups (P = 0 .53, P = 0.07 and P = 0.50, respectively, Table 2).

Birth weight of those infants who developed apnea in the control group (C) ranged from 600 to 1200 g (average birth weight: 989.37 g) and gestational age ranged from 25 to 32 wk (average gestational age: 27.81 wk). The time of occurrence of apnea ranged from the second to 10^th^ day of birth (median: 3 [3-5]). Table 3 presents the characteristics of infants who developed apnea.

**Table 1.** Characteristics of Study Infants ^a^

Characteristic	Aminophylline Group	Control Group	P Value
**Gestational age, wk**	29.89 ± 1.93	28.59 ± 2.06	0.10 ^b^
**Birth weight, g**	1071.54 ± 117.56	1007.69 ± 134.02	0.07 ^b^
**Gender **			0.58 ^c^
Male	13 (50)	11 (42.3)	
Female ^c^	13 (50)	15 (57.7)	

^a^ Data are presented as Mean ± SD and No. (%).

^b^ Independent t test.

^c^ Chi-Square Tests.

**Table 2.** Primary and Secondary Outcomes in our Study ^a^^,b^

Outcome	Aminophylline Group (A)	Control Group (C)	P Value
**n**	26	26	-
**Apnea**	2 (7.7)	16 (61.5)	< 0.001 ^c^
**Bradycardia**	3 (11.5)	16 (61.5)	< 0.001 ^c^
**Cyanosis**	4 (15.4)	16 (61.5)	< 0.001 ^c^
**CPAP [d, Median (IQR)]**	1 (0 - 4)	2.5 (0.5 - 6.5)	0.03 ^d^
**Mechanical ventilation**	1 (3.8)	0 (0)	0.50 ^d^
**Hospital stay [d, Median (IQR)]**	32.5 (23-53)	41 (27.5-50.5)	0.04 ^d^
**IVH**	0 (0)	3 (11.5)	0.07 ^c^
**PDA**	6 (23.1)	8 (30.8)	0.53 ^c^
**NEC**	3 (11.5)	2 (7.7)	0.50 ^e^
**Side effects**	0 (0)	0 (0)	> 0.999
**Death**	3 (11.5)	1 (3.8)	0.31^e^

^a^ Abbreviations: IVH; intraventricular hemorrhage, NEC; necrotizing enterocolitis, PDA; patent ductus arteriosus, CPAP; continuous positive airway pressure.

^b^The K-S (Kolmogorov-Smirnov) test showed that the distribution of quantitative variables was followed normal distribution. Data are presented as No. (%).

^c^ Chi-square.

^d^ Mann-Whitney test.

^e^ Fisher exact test.

**Table 3.** Characteristics of Neonates With Apnea ^a^

Group	GA, wk	BW, g	Gender	Age at Apnea Occurrence, d	CPAP, d	Age at Discharge, d
**A**	30	830	F	10	0	Death (14)
**A**	29	1100	F	3	1	Death (10)
**C**	30	1200	M	4	0	15
**C**	29	970	M	6	3	Death (18)
**C**	28	790	F	3	9	42
**C**	25	600	F	3	6	83
**C**	28	1100	M	3	6	30
**C**	26	910	M	3	10	54
**C**	28	1120	F	7	2	43
**C**	27	800	M	3	10	72
**C**	30	1120	F	2	2	39
**C**	28	1020	M	2	16	60
**C**	26	1000	M	3	4	37
**C**	26	1000	M	3	7	42
**C**	26	1000	F	3	6	41
**C**	27	1150	M	4	1	81
**C**	28+3d	1000	F	10	2	41
**C**	30	1050	F	9	4	31

^a^ Abbreviations: BW; birth weight, CPAP; continuous positive airway pressure, GA; gestational age.

## 5. Discussion

In our study, prophylactic aminophylline was effective in the reduction apnea incidence at higher-risk neonates (neonates with GA < 32 wk and/or BW < 1200 g). It seems that, the more premature an infant; the greater the preventative effect of aminophylline on the incidence of apnea and bradycardia. As mentioned in the introduction, a few studies have been done about preventive drugs for apnea of preterm infants. Now we present a brief review over studies and practices conducted on the prevention of neonates’ apnea.

Bucher et al. and Levitt et al. studied premature neonates (2, 7, 13). In each study, 20 mg/kg caffeine citrate as loading dose was used (2, 7, 13). Levitt used 5 mg/kg/day as the maintenance dose (2, 13), whereas Bucher and associates doubled that dose (7). Levitt continued his study until 32 weeks postmenstrual age, but Bucher continued his study up to 96 hours. In study of Bucher et al. bradycardia and hypoxia periods were reported as primary outcomes but was not reported apnea, directly (7). In the study of Levitt et al. apnea was reported as the primary outcome and described it as a pause of breathing for 20 s or more with cyanosis or bradycardia (HR < 100/min) (2, 13). In their studies, there was no difference between caffeine given group and placebo group with respect to primary outcomes (2, 7, 13). There was no difference in occurrence of side effects and mechanical ventilation use, too (2, 7, 13). 

But in our study, incidence of apnea was significantly lower in the aminophylline group compared to control group. This preventive effect of aminophylline in our study may be due to investigation of apnea incidence itself rather than hypoxemia and bradycardia (Bucher et al. study) (7), or due to clinical work at higher-risk neonates (preterm neonate with a BW < 1200 g). Another study, investigated the results such as death or inability in preterm neonates who have been given caffeine or placebo until corrected age of 18 to 21 months (3, 9-15). A subgroup of preterm infants who were qualified for inclusion in this large study was if they were been to be at risk of apnea of prematurity (3, 9-15). It was reported that the need to closure of PDA decreases in the group who had received prophylactic caffeine. Also the amount of time needed for mechanical ventilation with positive pressure had decreased, but they did not report the incidence of apnea (3, 9-15). The other results such as oxygen therapy periods, the amount of time needed for intubation, chronic lung disease (CLD), cerebral palsy, major inabilities or death showed no significant difference (3, 9-15).

Henderson-Smart DJ et al. after reviewing the relevant studies, reported that caffeine had no prophylactic effect for apnea of preterm infants (1). But they finally recommended that "Any future studies need to examine the effects of prophylactic methylxanthines in preterm infants at higher risk of apnea". This should include examination of important clinical outcomes such as need for IPPV (Intermittent Positive-Pressure Ventilation), neonatal morbidity, duration of hospital stay and long term development" (1).

Our study was planned to investigate the preventive effects of aminophylline on the incidence of neonatal apnea among higher-risk neonates (neonates with GA < 32 wk and/or BW < 1200 g). A large number of participated neonates in our study had RDS (Respiratory Distress Syndrome), but it did not affect primary and secondary outcomes. The results showed that, among extreme premature infants, the preventative effects of aminophylline on neonatal apnea are evident. Further investigation should, of course, be carried out to corroborate the findings of the present study.

The major limitation of this study could be the rather small number of the infants included (52 premature neonates), even though the results clearly indicated a statistically significant difference between the experimental and control groups. On the other hand, the direct supervision of study by a neonatologist and clinical trial at higher-risk neonates may be considered as the major strength of the study.

This study supports the preventative effects of aminophylline on the incidence of apnea in extreme premature infants. Actually, by studying these infants, preventative effects of aminophylline on neonatal apnea become apparent. In other words, the more premature an infant; the greater the preventative effect of aminophylline on the incidence of apnea and bradycardia.
